# Operative Technics

**Published:** 1914-03-15

**Authors:** 


### BIBLIOGRAPHY.

OPERATIVE TECHNICS. Under the title of Operative and Dental Anatomy Technics, P. Blokiston's Sons & Co., have just issued a new book on this subject by Dr. W. H. O. McGehee, Professor of Operative and Prothetic Dentistry, in the Ohio College of Dental Surgery. The book is intended to serve as an outline of preliminary or technic work which the dental student should do to fit him for taking up work in the clinic. The author has made free use of the material contributed to the subject in manuals, papers, etc., through the Institute of Dental Pedagogics and elsewhere. The student is also referred to the more elaborate consideration of the subject in the text books on Anatomy and Operative Dentistry. It is very fully illustrated with cuts and original drawings and is in every way a most satisfactory presentation of the subject. It takes up every technical procedure from the study of the anatomy of the tooth to the treatment of the pulp canal for introduction of the filling material. It is so completely and well done that very little criticism can be made. In fact we find nothing to criticise and lots to conview. We predict this little book will become one of our standard student helps. We congratulate the Author in his comprehension and satisfactory presentation of the subject.

